# A high blood endocan profile during COVID-19 distinguishes moderate from severe acute respiratory distress syndrome

**DOI:** 10.1186/s13054-021-03589-3

**Published:** 2021-05-06

**Authors:** Tiffany Pascreau, Colas Tcherakian, Benjamin Zuber, Eric Farfour, Marc Vasse, Philippe Lassalle

**Affiliations:** 1 Department of CLINICAL Biology, Foch Hospital, 40 rue Worth, 92150 Suresnes, France; 2 INSERM UMR_S 1176, University Paris-Sud, University Paris-Saclay, Le Kremlin-Bicêtre, France; 3 Department of Pneumology, Foch Hospital, Suresnes, France; 4 Department of Medical and Surgical Intensive Care Unit, Foch Hospital, Suresnes, France; 5 Univ. Lille, CNRS, Inserm, CHU Lille, Institut Pasteur de Lille, U1019-UMR9017-CIIL-Centre d’Infection et d’Immunité de Lille, Equipe immunité pulmonaire, Lille, France; 6 Biothelis, 59000 Lille, France

Endocan is expressed by the lung endothelial cells and upregulated by pro-inflammatory conditions. Because elevated pro-inflammatory cytokines are hallmarks of severe SARS-CoV-2 infection [1], and that the lung is the organ preferentially affected, we measured endocan in patients with COVID-19 pneumonia. This retrospective study was conducted between March 12 and April 20, 2020, and approved by the local ethics committee of the Foch Hospital (20-07-15). Seventy-four patients with COVID-19 pneumonia confirmed by RT-PCR were enrolled. According to the Berlin definition of acute respiratory distress syndrome (ARDS), patients were categorized into non-ARDS, mild/moderate ARDS, and severe ARDS. At the admission complete blood count, biochemical and coagulation parameters were measured. Endocan and cathepsin G-cleaved endocan (p14) concentrations were measured on baseline and during the hospitalization. Plasmatic endocan cleavage ratio (ECR) was calculated as plasma p14/(endocan + p14) ratio. Healthy hospital workers served as group of control. Baseline characteristics and clinical outcomes are summarized in Table 1. Patients with ARDS had significantly higher CRP (176 mg/L [IQR: 133–270] vs 141 mg/L [IQR: 88–187], *p* = 0.0122), and higher d-dimers (1.843 mg/L [IQR: 0.579–7.134] vs 0.771 mg/L [IQR: 0.535–1.374], *p* = 0.0472), had greater lung parenchyma involvement assessed by the CT score (4 [4, 5] vs 3 [2–4], *p* = 0.0016) and stay hospitalized for longer than non-ARDS group (25 days [IQR: 14–38] vs 12 days [IQR: 7–17], *p* < 0.0001). The mortality rate was also higher in patients with ARDS than non-ARDS group (43% vs 5%, *p* < 0.0001) (Table 1). At admission, endocan levels measured in 59/74 (84%) patients were significantly increased in patients with COVID-19 compared to controls (3.4 ng/mL [IQR: 1.8–7.5] vs 1.6 ng/mL [IQR: 1.0–2.1], respectively, *p* = 0.0031) (Fig. 1a). There was no significant difference between patients who developed ARDS and those who have not (3.7 [2.8–9.6] ng/mL vs 3.2 [1.5–5.7] ng/mL, respectively, *p* = 0.2231) (Fig. 1b). Endocan was negatively correlated with the platelets (Spearman's correlation coefficient *r* = − 0.3681, *p* = 0.0041). When plasma samples were available in patients with ARDS, endocan concentrations were measured during the hospitalization. Patients with mild/moderate ARDS had a significant increase in endocan levels at days 3–4 (*p* = 0.0084) and days 5–6 (*p* = 0.0107) compared to those measured at days 1–2 (Fig. 1c). No increase was observed in patients with severe ARDS (Fig. 1c). This discrepancy was not due to an increase in cleavage of endocan since the ECR remained similar whatever the severity of ARDS or the hospitalization day (not shown). ECR at admission was positively correlated with the Von Willebrand antigen (*r* = 0.3047, *p* = 0.0418). ARDS was already present at admission in 15/37 (40%). Twelve (32%) patients developed ARDS within 48 h, whereas 10 (27%) patients developed ARDS between the 3rd and 7th day of hospitalization. After exclusion of patients with ARDS within the first 48 h, the calculated AUC of endocan was 0.7235 (*p* = 0.1104). Several biomarkers had already been related to the ARDS [2, 3]. In a series of 659 patients with COVID-19, lymphocyte count, creatine kinase, neutrophils/lymphocytes ratio, AST, lactate dehydrogenase, and CRP were all strongly related to the aggravation of ARDS [4]. Few data are available in the literature about endocan in COVID-19. One study observed that endocan levels at the admission were associated with poor clinical outcomes, but the occurrence of ARDS has not been studied [5]. In our series, endocan levels measured at admission could be predictive of ARDS after the 3rd day of hospitalization. During the hospitalization, we observed a lack of increase in endocan levels in patients with severe ARDS which is consistent with the finding that low endocan levels during sepsis may also predict ARDS worsening [6]. Further prospective studies are required to confirm these results on the role of endocan in prediction of ARDS in patients with COVID-19.

**Table 1:** Baseline characteristics, biological and radiological findings at the admission and clinical outcomes of the patients

	All (*n* = 74)	Non-ARDS (*n* = 37)	ARDS (*n* = 37)	ARDS vs non-ARDS	Mild or moderate ARDS (*n* = 12)	Severe ARDS (*n* = 25)	Non-ARDS vs mild/moderate vs severe ARDS
Male sex, *n* (%)	59 (80%) (*n* = 74)	30 (81%) (*n* = 37)	29 (78%)	> 0.9999	9 (75%)	20 (80%)	0.9008
Age, median [IQR] (years)	64 [55–71] (*n* = 74)	65 [51–73] (*n* = 37)	62 [56–70]	0.8486	63 [56–76]	62 [55–68]	0.6131
BMI (kg/m2)	26.9 [24.0–31.0] (*n* = 53)	26.5 [23.7–30.6] (*n* = 30)	27.3 [24.2–31.0] (*n* = 23)	0.4355	24.2 [23.4–32.7] (*n* = 7)	27.7 [25.4–30.6] (*n* = 16)	0.5424
*Comorbidities*							
Hypertension *n* (%)	36 (49%) (*n* = 74)	19 (63%)	17 (46%)	0.8163	5 (42%)	12 (48%)	0.8408
Diabetes *n* (%)	31 (42%) (*n* = 74)	14 (47%)	17 (46%)	0.6378	7 (58%)	10 (40%)	0.4450
*Biological and radiological parameters at admission*							
CRP (mg/L) *N* < 5 mg/L	155 [109–217] (*n* = 70)	141 [88–187] (*n* = 34)	176 [133–270] (*n* = 36)	**0.0122**	178 [125–301] (*n* = 12)	175 [133–267] (*n* = 24)	**0.0447**
Creatinine (µmol/L) *N *49–90 (F); 64–104 (M) µmol/L	95 [70–131] (*n* = 74)	100 [74–132] (*n* = 37)	93 [67–141] (*n* = 37)	0.4794	98 [65–127] (*n* = 12)	93 [68–165] (*n* = 25)	0.7714
AST (UI/L) *N* 5–34 UI/L	63 [42–88] (*n* = 70)	57 [40–84] (*n* = 36)	72 [43–98] (*n* = 34)	0.1550	65 [39–111] (*n* = 10)	77 [45–90] (*n* = 24)	0.3262
ALT (UI/L) *N* < 55 UI/L	32 [21–52] (*n* = 70)	28 [21–50] (*n* = 36)	34 [23–53] (*n* = 34)	0.4846	40 [12–53] (*n* = 10)	34 [23–60] (*n* = 24)	0.8455
Ferritin (µg/L) *N* 5–204 (F); 22–275 (M) µg/L	1522 [770–2688] (*n* = 51)	1183 [574–2291] (*n* = 24)	1602 [1068–2704] (*n* = 27)	0.1773	1252 [599–2531] (*n* = 8)	2064 [1104–4130] (*n* = 19)	0.1966
Platelets (10^9^/L) *N* 150–450 10^9^/L	189 [143–244] (*n* = 73)	186 [143–244] (*n* = 37)	191 [142–252] (*n* = 36)	0.8242	205 [152–268] (*n* = 12)	182 [130–229] (*n* = 24)	0.6761
PNN (10^9^/L) *N* 1.5–7.5 10^9^/L	6.4 [4.3–8.8] (*n* = 73)	6.1[4.1–7.6] (*n* = 37)	7.0 [4.5–9.0] (*n* = 36)	0.0750	8.1 [4.4–9.0] (*n* = 12)	7.0 [4.5–9.5] (*n* = 24)	0.1857
VWF antigen (%) *N* 50–150%	429 [331–498] (*n* = 64)	347 [299–509] (*n* = 35)	449 [368–494] (*n* = 29)	0.1648	476 [403–546] (*n* = 10)	446 [331–484] (*n* = 19)	0.2497
Fibrinogen (g/L) *N* 1.5–3.5 g/L	6.10 [4.15–6.82] (*n* = 52)	5.14 [4.03–6.46] (*n* = 28)	6.32 [4.37–7.13] (*n* = 24)	0.1056	6.40 [6.14–7.11] (*n* = 8)	6.18 [4.24–7.13] (*n* = 16)	0.2153
D-dimers (mg/L) *N* < 0.500 mg/L	1.079 [0.538–3.607] (*n* = 61)	0.771 [0.535–1.374] (*n* = 31)	1.843 [0.579–7.134] (*n* = 30)	**0.0472**	1.492 [0.569–3.228] (*n* = 10)	1.843 [0.586–17.64] (*n* = 20)	0.1207
Initial CT findings (score)*	4 [3–4] (*n* = 39)	3 [2–4] (*n* = 22)	4 [4–5] (*n* = 17)	**0.0016**	4 [4–5] (*n* = 9)	4 [4–5] (*n* = 6)	**0.0171**
*Clinical* *outcomes*							
Time from illness onset to hospital admission (days)	8 [6–12] (*n* = 69)	9 [7–12] (*n* = 34)	8 [5–12] (*n* = 37)	0.7217	8 [4–10] (*n* = 11)	8 [5–12] (*n* = 24)	0.5818
Time from illness onset to occurrence of ARDS	–	–	10 [8–14] (*n* = 32)	–	8 [5–14] (*n* = 9)	11 [8–15] (*n* = 23)	0.1711
Hospital stay (days)	16 [10–29] (*n* = 73)	12 [7–17] (*n* = 36)	25 [14–38] (*n* = 35)	** < 0.0001**	23 [14–30] (*n* = 12)	27 [14–41] (*n* = 25)	**0.0004**
Mortality *n* (%)	18 (24%) (*n* = 74)	2 (5%) (*n* = 37)	16 (43%) (*n* = 37)	**0.0001**	3 (25%) (*n* = 12)	13 (52%)	**0.0002**

^*^Semiquantitative CT score was calculated based on the extent of lung parenchyma involvement (1: 0–10%; 2: 11–25%; 3: 26–50%; 4: 51–75%; 5:  > 75%)

In univariate analysis, we determined the differences in median using unpaired t test (Mann–Whitney U test) for continuous variable and differences in proportions were determined using the Chi-square test or Fischer exact test, as appropriate. Concentrations of endocan were compared between groups (control, non-ARDS, mild/moderate ARDS and severe ARDS) using the Kruskal–Wallis test followed by Dunn’s posttest. Bold font indicates statistical significance

*AST* aspartate aminotransferase, *ALT* alanine aminotransferase, *PNN* polynuclear neutrophils, *VWF* von Willebrand factor

**Fig. 1:**
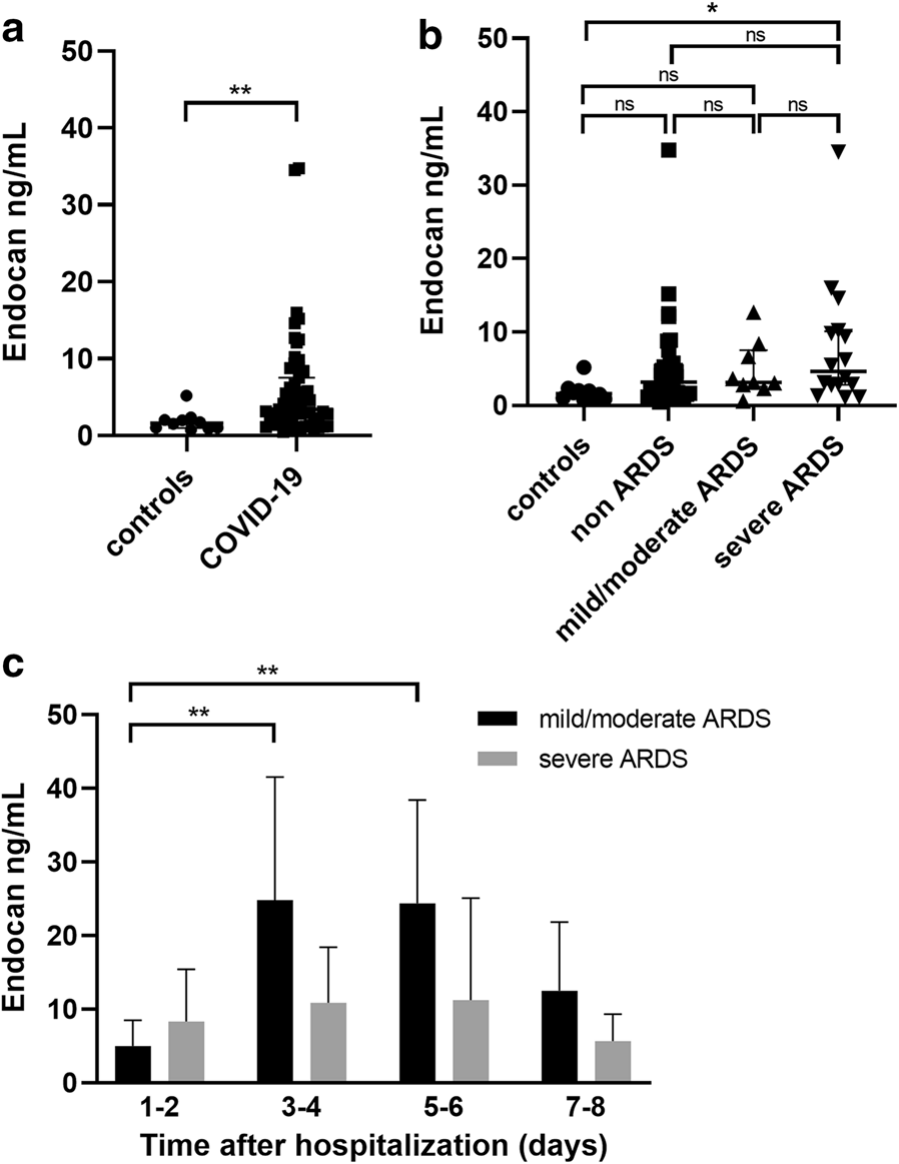
Endocan concentrations in patients with COVID-19 at admission and during the hospitalization. **a** Endocan levels measured at the admission in patients with COVID-19. Data are presented as median and interquartile range. Patients with COVID-19 were compared to the control group using the Mann–Whitney test. *p < 0.05. **b** Endocan levels measured at the admission in patients with COVID-19 categorized into non-ARDS, mild/moderate ARDS and severe ARDS. Data are presented as median and interquartile range. Results were expressed as mean and standard deviation. Intergroup comparison was made using the Kruskal–Wallis test. *p < 0.05. **c** Endocan levels measured over the course of hospitalization in patients with COVID-19 and ARDS. Mild/moderate ARDS group *n* = 10 (days 1–2), *n* = 3 (days 3–4), *n* = 3 (days 5–6), *n* = 2 (days 7–8); severe ARDS group *n* = 18 (days 1–2), *n* = 12 (days 3–4), *n* = 13 (days 5–6), *n* = 9 (days 7–8). Results are expressed as mean ± standard deviation. Difference in endocan levels over the course of the hospitalization was calculated using the two-way ANOVA followed by Bonferroni’s posttest. **p < 0.01

## Data Availability

The datasets analyzed during the current study are available from the corresponding author on reasonable request.

## Abbreviations

ARDS: Acute respiratory distress syndrome
AST: Aspartate aminotransferase
AUC: Area under curve
COVID-19: Coronavirus disease 2019
CRP: C-reactive protein
CT: Computed tomography
ECR: Endocan cleaved ratio
IQR: Interquartile range
